# Mobile Health Interventions Across the Stroke Care Continuum: A Scoping Review

**DOI:** 10.3390/jcm15114121

**Published:** 2026-05-26

**Authors:** Dahyeon Koo, Seunggyun Jeong, Kyumin Jang, Younghwan Jang, Seo Yeong Bae, Soonmi Kwon, Dougho Park

**Affiliations:** 1 Medical Research Institute, Pohang Stroke and Spine Hospital, Pohang 37659, Republic of Korea; dahyeonkoo@gmail.com (D.K.); radical529@gmail.com (K.J.); 2 Healthbuddy Co., Ltd., Pohang 37673, Republic of Korea; jskyun98@gmail.com; 3 Department of Physical Therapy, Pohang Stroke and Spine Hospital, Pohang 37659, Republic of Korea; jang1041109@gmail.com (Y.J.);; 4 Department of Occupational Therapy, Pohang Stroke and Spine Hospital, Pohang 37659, Republic of Korea; 5 Medical Science and Engineering, Graduate School of Convergence Science and Technology, Pohang University of Science and Technology, Pohang 37666, Republic of Korea

**Keywords:** digital health, mHealth, scoping review, stroke

## Abstract

Stroke causes approximately 12.2 million new cases and 6.5 million deaths annually, with survivors requiring coordinated care across pre-hospital, acute, rehabilitative, and preventive phases. Mobile health (mHealth) technologies, including smartphone applications, wearable sensors, and tablet-based platforms, have shown clinical potential across these contexts, yet a structured mapping of their distribution across the full stroke care continuum is lacking. We searched PubMed, Scopus, and Web of Science for publications from January 2019 to March 2025. Studies evaluated mHealth interventions in which the mobile platform directly performed diagnostic, therapeutic, or rehabilitative functions in stroke populations. Of 4524 records identified, 17 met the inclusion criteria. Studies originated from eight countries and used heterogeneous designs: five randomized controlled trials, five non-randomized studies, four cohort studies, and three diagnostic accuracy studies. Median sample size was 37 participants (range 10–2249). Evidence concentrated at two poles: six studies addressed acute diagnosis and ten addressed rehabilitation, predominantly in the chronic phase. One study addressed secondary prevention; two targeted early rehabilitation, the period of maximum neuroplasticity after discharge. All seventeen studies covered a single care phase. Smartphone platforms dominated acute contexts; wearable and mixed-modality systems were confined to rehabilitation. The mHealth stroke landscape is fragmented and phase-specific, exhibiting a silo effect in which interventions operate as isolated tools rather than components of an integrated care system. An important gap is the near-absence of research in early rehabilitation. Future priorities include cross-continuum design, expansion into cognitive and secondary prevention domains, and progression toward adequately powered trials.

## 1. Introduction

Stroke is one of the leading causes of death and long-term disability worldwide. According to the Global Burden of Disease Study 2019, stroke accounted for approximately 12.2 million new cases and 6.5 million deaths annually, making it the second most common cause of mortality and the third most common cause of disability-adjusted life years globally [1]. Beyond its acute consequences, stroke survivors frequently experience persistent motor, cognitive, and communicative impairments, necessitating continuous and multidisciplinary care extending well beyond the acute hospitalization phase [2].

The concept of the stroke care continuum captures the sequential and interconnected nature of stroke management, encompassing pre-hospital recognition and triage, acute in-hospital diagnosis and treatment, early rehabilitation, and long-term chronic phase management, including secondary prevention [3,4]. Effective care across this continuum is critical to minimizing disability and reducing the risk of recurrence [5]. Yet, delivery of care at each stage presents distinct challenges, including time-sensitivity in the acute phase, accessibility constraints during rehabilitation, and the need for sustained behavioral change in secondary prevention.

Conventional stroke rehabilitation primarily relies on therapist-supervised physical therapy, occupational therapy, and speech-language therapy delivered in inpatient or outpatient settings. These approaches provide individualized assessment, direct clinician feedback, and structured multidisciplinary care. However, access to long-term rehabilitation is often limited by resource availability, transportation burden, cost, and reduced continuity of care after hospital discharge. Mobile health (mHealth) technologies—including smartphone-based rehabilitation applications, wearable motion sensors for gait and balance monitoring, and tablet-based speech or cognitive rehabilitation platforms—have emerged as promising tools to address these challenges [6]. By enabling real-time monitoring, remote communication, and personalized intervention delivery, mHealth has demonstrated potential across diverse clinical contexts: supporting emergency triage and rapid diagnostic decision-making in the acute phase, facilitating home-based rehabilitation in the subacute and chronic phases, and promoting medication adherence and lifestyle modification for secondary prevention [7,8]. A growing body of evidence, including systematic reviews and meta-analyses, has reported favorable effects of digital interventions on post-stroke recovery outcomes [6,7].

Despite this progress, the existing literature on mHealth for stroke care has been largely domain-specific. Reviews have focused on discrete segments of care, such as digital rehabilitation [6], telemedicine-based acute stroke management [8], or post-stroke self-management interventions [9]. While these targeted reviews have provided valuable insights within their respective domains, they do not offer a comprehensive picture of how mHealth interventions are distributed and applied across the full stroke care continuum [10,11]. As a result, important structural features of the mHealth landscape—including the presence of research gaps between care phases and the degree of fragmentation across the continuum—remain insufficiently characterized [12].

Furthermore, although prior reviews have addressed mHealth interventions within specific phases of stroke care, few have offered a more explicit, structured continuum mapping that classifies interventions by stage of application and visualizes the resulting distribution using an evidence map. Such an integrative synthesis would be particularly valuable for identifying underserved areas of the continuum and informing the design of more integrated mHealth solutions [13].

Therefore, this scoping review aimed to map the distribution of mHealth interventions across the stroke care continuum and to identify research gaps and priorities for future development. Specifically, the review sought to: (1) systematically identify and describe mHealth interventions applied across the stroke care continuum; (2) characterize the distribution of these interventions by care stage, intervention type, and technology modality; and (3) generate an evidence map to visualize the relationship between intervention types and clinical domains, thereby identifying research gaps and priorities for future development.

## 2. Materials and Methods

### 2.1. Protocol and Registration

This study was conducted as a scoping review following the Preferred Reporting Items for Systematic Reviews and Meta-Analyses extension for Scoping Reviews (PRISMA-ScR) guidelines [14]. The completed PRISMA-ScR checklist is provided as Supplementary Material. The review was conducted in accordance with a pre-defined search strategy and eligibility criteria established before the screening process commenced.

### 2.2. Eligibility Criteria

Studies were eligible for inclusion if they met all of the following criteria: (1) original analytical articles evaluating the effectiveness or efficacy of an intervention, including randomized controlled trials (RCTs), observational studies, and diagnostic accuracy studies; (2) mHealth-based interventions involving mobile applications, wearable devices, or tablet-based platforms; (3) interventions in which the mobile application or wearable device directly performed or delivered diagnostic, therapeutic, rehabilitative, or decision-making functions, rather than serving solely as a support or communication tool; (4) study populations including patients with ischemic or hemorrhagic stroke; and (5) full-text articles published in English.

Studies were excluded if: (1) they were non-original publications; (2) they assessed only technological development or feasibility without evaluating clinical effectiveness or diagnostic accuracy; (3) they evaluated web-based or telemedicine systems in which mobile devices were used primarily for communication, monitoring, or data transmission; (4) the mobile application was used only as a delivery or support tool (e.g., for reminders, education, data collection, or workflow coordination) without directly performing diagnostic or therapeutic functions; or (5) stroke-specific outcomes could not be extracted.

Accordingly, this review focused on functionally active mHealth interventions directly involved in assessment, monitoring, decision support, or rehabilitation, rather than passive communication- or reminder-based tools.

### 2.3. Information Sources

A systematic literature search was conducted across three electronic databases: PubMed, Scopus, and Web of Science. The search was restricted to publications from January 2019 to March 2025, to capture contemporary mHealth developments and align with the rapid evolution of wearable and application-based technologies in clinical practice. Database searches were performed using a Python-based Application Programming Interface.

### 2.4. Search Strategy

The search strategy combined stroke-related and digital health-related terms. Stroke-related terms included: Stroke, Cerebral Infarction, Intracranial Hemorrhage, and Subarachnoid Hemorrhage. Digital health-related terms included: mHealth, eHealth, Telemedicine, and Digital Health. The final search string was:

(Stroke OR Cerebral Infarction OR Intracranial Hemorrhage OR Subarachnoid Hemorrhage) AND (mHealth OR eHealth OR Telemedicine OR Digital Health).

In PubMed, these free-text terms correspond to the following MeSH descriptors: Stroke, Cerebral Infarction, Intracranial Hemorrhages, Subarachnoid Hemorrhage, and Telemedicine (which serves as the PubMed MeSH term encompassing mHealth, eHealth, and related digital health delivery concepts). Digital Health was added to MeSH in 2024.

This strategy was intentionally broad to maximize sensitivity. We acknowledge that the exclusion of terms such as “mobile application,” “smartphone app,” or “app-based” may have resulted in the omission of some relevant studies; this limitation is addressed in the Section 5.

### 2.5. Selection of Sources of Evidence

A total of 4524 records were initially identified. Following exclusion of 161 records with missing digital object identifiers (DOIs), duplicate removal based on DOI matching yielded 2384 unique records for screening.

Title and abstract screening were performed by seven reviewers using a standardized Google Spreadsheet-based protocol. Records were divided into eight contiguous parts of approximately equal size (A–H), and each reviewer was assigned two adjacent parts, with neighboring reviewers sharing one part. This arrangement resulted in each reviewer being responsible for approximately one-quarter of the total records, while approximately 75% of records (1789 of 2384) were independently reviewed by two reviewers. In the overlap zone, raw inter-reviewer agreement was 96.3%. For records with discordant judgments, the two reviewers re-evaluated the case to reach consensus; cases without consensus were adjudicated by three reviewers who co-authored this study (D.K., S.J., and D.P.).

**Figure 1:**
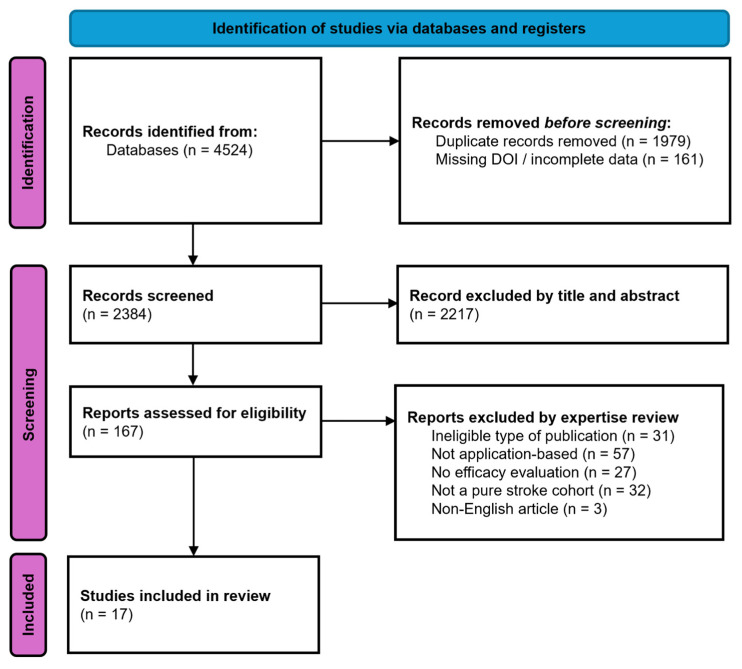
PRISMA-ScR flow diagram.

A total of 167 records advanced to full-text review. Following full-text evaluation, 150 records were excluded for the following reasons: non-original study design (*n* = 31), mHealth component not meeting eligibility criteria (*n* = 57), descriptive study or technology development without efficacy evaluation (*n* = 27), mixed neurological population without extractable stroke-specific data (*n* = 32), and non-English articles (*n* = 3). Finally, a total of 17 studies were included in this review (Figure 1).

### 2.6. Data Charting Process and Items

Data extraction was performed independently by two reviewers using a pre-defined charting form. The three reviewers (D.K., S.J., and D.P.) adjudicated discrepancies. Prior to the main extraction phase, the charting form was piloted on a random sample of five included studies and refined accordingly.

The following variables were extracted from each included study: (1) publication year; (2) country; (3) stroke stage (pre-hospital, acute, early rehabilitation, chronic, post-acute, or mixed); (4) stroke type (ischemic, hemorrhagic, or both); (5) care domain (acute diagnosis, rehabilitation, secondary prevention, or management/recovery support); (6) study design; (7) sample size; (8) technology type (smartphone, wearable device, tablet, or mixed); and (9) application or platform name. Each study was additionally classified by its position along the stroke care continuum (pre-hospital, in-hospital, post-hospital) and by the primary purpose of the intervention (diagnosis, treatment, or prevention).

### 2.7. Critical Appraisal of Individual Sources of Evidence

In keeping with scoping review methodology, which is designed to map the breadth and distribution of available evidence rather than to appraise its quality, formal quality assessment or risk of bias evaluation of individual studies was not conducted. The heterogeneity in study designs across included studies, ranging from RCTs and diagnostic accuracy studies to observational cohorts, is consistent with the exploratory nature of this review.

### 2.8. Synthesis of Results

Extracted data were organized and analyzed descriptively. Studies were classified according to stroke care continuum phase, intervention type, and technology modality, and frequencies and proportions were calculated for each category. An evidence map was constructed as a bubble plot, with intervention type on the x-axis and clinical domain on the y-axis; bubble size reflected the number of studies within each cell. All descriptive analyses and figures, including the evidence map, were performed in Python 3.13.2 using the pandas (v3.0.1), NumPy (v2.4.3), and matplotlib (v3.10.8) libraries. This approach facilitates identification of research concentration and gaps across the continuum.

## 3. Results

### 3.1. Study Selection and Overview

A total of 17 studies published between 2020 and 2025 were included in this scoping review (Table 1). The included studies originated from eight countries: the United States (*n* = 8), Brazil (*n* = 2), Spain (*n* = 2), Japan (*n* = 1), Chile (*n* = 1), Egypt (*n* = 1), China (*n* = 1), and Canada (*n* = 1). This geographic distribution suggests a concentration of mHealth stroke research in high-income countries, with relatively limited representation from low- and middle-income settings. Publication frequency increased progressively over the review period, with the majority of studies (*n* = 7) published in 2024 or 2025, suggesting accelerating research activity in this domain.

Study designs were heterogeneous. A total of 10 interventional studies: five RCTs and five non-randomized studies. Seven observational studies consisted of diagnostic accuracy studies (*n* = 3) and cohort studies (*n* = 4). Sample sizes ranged widely, from 10 to 2249 participants; however, most studies enrolled fewer than 100 participants, indicating that the current evidence base is largely derived from small-scale investigations. Notably, four studies included fewer than 15 participants, highlighting the continued prevalence of pilot and feasibility studies in this field.

### 3.2. Distribution Across the Stroke Care Continuum

**Figure 2:**
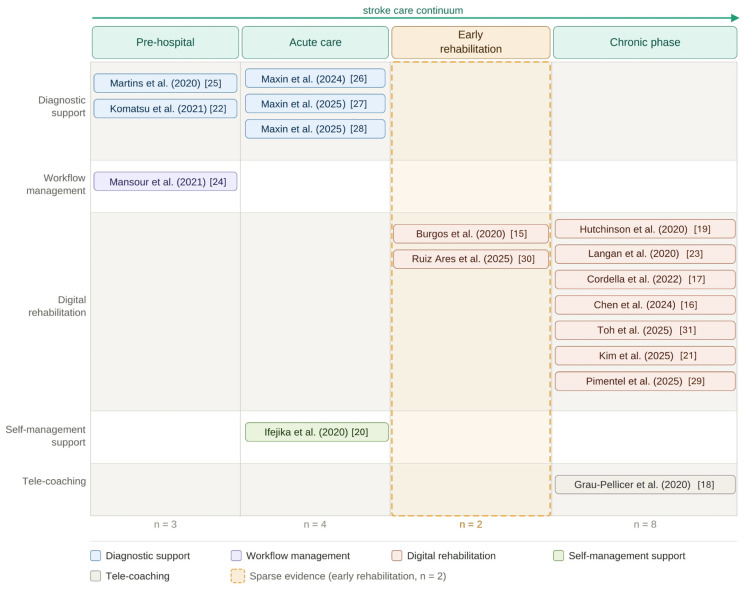
Distribution of mHealth interventions across the stroke care continuum. Each column represents a phase of the stroke care continuum, progressing from left (pre-hospital) to right (chronic phase). Each row represents an intervention type. Labelled pills indicate individual included studies assigned to the corresponding phase–intervention combination based on the primary timing and functional classification of the mHealth platform. The amber-bordered column (early rehabilitation) highlights the phase with the fewest studies (*n* = 2), reflecting a relative research gap in this transitional period despite its recognized importance for neurological recovery. Study counts below each column represent the total number of studies classified within that phase [15,16,17,18,19,20,21,22,23,24,25,26,27,28,29,30,31].

When mapped across the stroke care continuum, the distribution of mHealth interventions was uneven across phases of care (Figure 2). The largest number of studies was concentrated in the chronic phase, followed by the acute care phase, whereas the early rehabilitation phase was represented by only two studies. The pre-hospital phase was addressed by three studies, although all were limited to diagnostic support or workflow-related functions rather than therapeutic or rehabilitative applications.

In the pre-hospital phase, the identified interventions focused primarily on early stroke recognition, triage, and coordination of care. Acute care studies were also largely oriented toward diagnostic support, including application-assisted tools designed to support clinical decision-making and imaging interpretation, with only limited representation of self-management support. In contrast, the early rehabilitation phase was represented exclusively by digital rehabilitation interventions.

The chronic phase accounted for the greatest concentration of studies and was dominated by rehabilitation-focused applications. Most interventions in this phase were classified as digital rehabilitation, frequently incorporating wearable or sensor-based systems to support motor recovery in post-stroke populations, while only one study was categorized as tele-coaching. Notably, no study was designed to span multiple stages of the stroke care continuum as a primary focus, indicating that current mHealth interventions remain largely phase-specific rather than integrated across the broader trajectory of stroke care.

### 3.3. Technology Modalities and Care Domains

The technology modalities employed varied across care domains (Table 2). Smartphone-based applications were the most commonly used platform and were predominant in acute diagnosis and secondary prevention, reflecting the need for portability and rapid accessibility in time-sensitive and scalable clinical contexts.

In contrast, rehabilitation-focused interventions employed a more diverse range of technologies, including wearable devices, smartphones, tablets, and hybrid systems. Wearable devices were used exclusively within rehabilitation contexts, where they served as motion-sensing platforms to support motor training and functional assessment. These systems frequently incorporated sensor-based feedback mechanisms, enabling more continuous and ecologically relevant monitoring of motor performance.

This modality-by-domain pattern reflects the functional requirements of each stage of care. In acute settings, smartphone-based tools facilitate rapid clinical decision-making, whereas in rehabilitation, the use of wearable technologies supports ongoing monitoring and interactive feedback during recovery.

### 3.4. Evidence Map of Intervention Types and Clinical Domains

**Figure 3:**
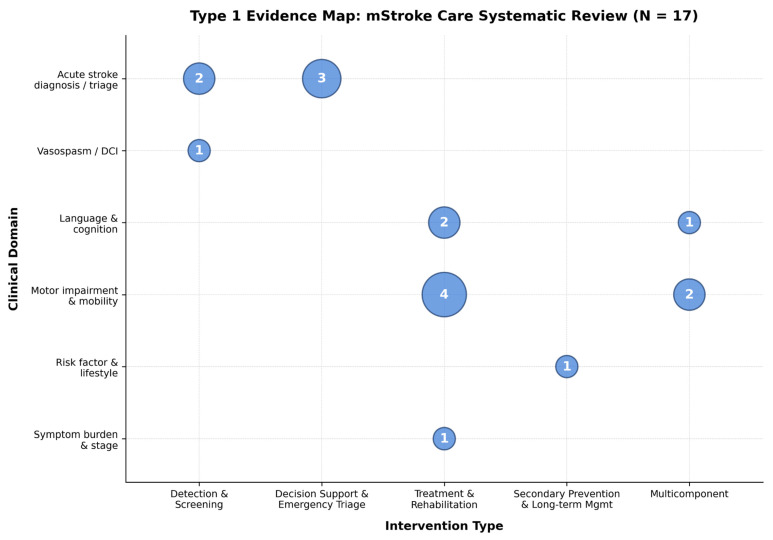
Evidence map of mobile health interventions for stroke care. The x-axis represents the functional category of the mHealth intervention, and the y-axis represents the primary clinical domain addressed. Each bubble corresponds to a cell in the domain–function matrix; bubble size is proportional to the number of included studies classified within that combination, with the total study count labeled inside each bubble. Cells without a bubble indicate domain–function combinations for which no study was identified. The largest cluster, motor impairment and mobility × treatment and rehabilitation, reflects the dominance of chronic-phase motor rehabilitation in the current evidence base. Sparsely populated cells in the language and cognitive function, risk factor, and lifestyle management domains highlight clinically important but underrepresented areas of mHealth stroke research.

The evidence map (Figure 3) illustrates the distribution of mHealth interventions across intervention types and clinical domains. A clear clustering pattern was observed, with interventions concentrated in specific combinations of domains and functions.

Interventions targeting acute stroke diagnosis and triage were primarily associated with detection and screening, as well as decision support and emergency triage functions. In contrast, interventions addressing motor impairment and mobility were predominantly categorized under treatment and rehabilitation, representing the largest cluster within the map.

Other clinical domains were sparsely represented. Interventions targeting language and cognitive function were distributed across rehabilitation and multicomponent approaches, while risk factor and lifestyle management were linked exclusively to secondary prevention and long-term management. Additionally, symptom burden and stroke stage-related outcomes were minimally represented, appearing only within the rehabilitation category.

Overall, the evidence map highlights a concentration of mHealth interventions in a limited set of domain–function combinations, with relatively few studies addressing broader or more diverse clinical domains.

## 4. Discussion

This scoping review systematically mapped mHealth interventions across the stroke care continuum and identified a distinctly uneven and fragmented distribution of evidence. Three structural patterns emerged from the data. First, the evidence base is concentrated at two poles—acute diagnosis and chronic rehabilitation—while intermediate phases of care remain substantially underrepresented. Second, technology modality is closely aligned with care domain: smartphone-based applications predominate in acute diagnostic contexts, while wearable and mixed-modality systems are confined to rehabilitation settings. Third, the overall evidence base remains methodologically immature, with a median sample size of 37 participants across all included studies, thirteen of seventeen studies enrolling fewer than 50 participants, and four studies enrolling fewer than 15.

A central structural finding is the lack of included studies of cross-continuum integration. All seventeen included studies addressed a single phase of care, with no intervention designed to span multiple stages of the continuum. This phase-specific design creates a structural mismatch with the longitudinal reality of stroke care, which requires coordinated management from emergency recognition through acute treatment, early recovery, and long-term secondary prevention. Current mHealth interventions thus function as isolated, phase-bound tools rather than as components of a continuous and integrated care system—a pattern we term the silo effect in digital stroke care.

The concentration of mHealth research in acute stroke diagnosis is clinically coherent. Six of the seventeen included studies addressed acute diagnosis, spanning pre-hospital recognition [22,24,25] and in-hospital diagnostic support [26,27,28]. In the hyperacute setting, time-to-treatment is the principal determinant of neurological outcome, and smartphone-based platforms offer a portable and rapidly deployable mechanism for image sharing, triage coordination, and clinical decision support. The three diagnostic accuracy studies provide a methodologically rigorous foundation for this phase, demonstrating that smartphone-assisted imaging review can achieve near-equivalent diagnostic performance to conventional workstation-based assessment for large vessel occlusion detection and stroke classification [22,26,28].

Pre-hospital studies addressed diagnostic support and workflow coordination exclusively, with no patient-facing rehabilitative or educational component, reflecting the time-critical constraints of emergency medicine [22,24,25]. The practical implication for acute care clinicians is clear: smartphone-based diagnostic tools represent the most mature and immediately deployable mHealth application in stroke care. However, they address only one dimension of the care needs that arise in the critical early hours after stroke onset, and the evidence base does not yet extend to patient-centered applications in the pre-hospital phase.

The chronic phase accounted for eight of the seventeen included studies—the single largest concentration of evidence—all of which targeted rehabilitation. This dominance reflects both the clinical magnitude of post-stroke disability and the practical feasibility of deploying mHealth interventions in community settings, where patients have sufficient time and cognitive capacity for sustained technology engagement. The technology diversity in this phase is notable: smartphone-based platforms were employed by Chen et al. [16], Hutchinson et al. [19], Langan et al. [23], and Kim et al. [21]; a wearable device was used by Toh et al. [31]; mixed-modality systems integrating motion-sensing wearables with smartphone interfaces were employed by Burgos et al. [15] and Pimentel et al. [29]; and a tele-coaching application was evaluated by Grau-Pellicer et al. [18]. This diversity indicates a field actively exploring which technology configurations best support the multidimensional demands of post-stroke recovery. However, the evidence quality in the chronic phase is uneven. The eight chronic-phase studies enrolled a median of 20 participants, and four studies enrolled fewer than 20 participants each, precluding meaningful inference about clinical effectiveness. Furthermore, the therapeutic targets are narrow: motor rehabilitation dominated, and cognitive, speech, and behavioral domains were addressed by only a minority of studies. Given that post-stroke cognitive impairment affects up to 60% of stroke survivors [32], and post-stroke depression affects up to 28% [33], the underrepresentation of these domains represents a clinically significant gap in the current mHealth literature.

The most clinically consequential finding of this review is the near-absence of mHealth research in the early rehabilitation phase—the period spanning from hospital discharge to community reintegration, typically encompassing the first weeks to months after stroke onset. Only two of 17 studies explicitly targeted this phase: Burgos et al. [15], who evaluated an exergame-based smartphone system for balance rehabilitation in the early subacute period, and Ruiz Ares et al. [30], who assessed a digital speech rehabilitation tool in patients with acute-to-subacute aphasia. Together, these two studies represent just 11.8% of the included evidence base, despite the early rehabilitation phase being the period of maximum neuroplasticity, when intensive, task-specific rehabilitation carries the greatest potential to promote functional recovery through experience-dependent cortical reorganization [34,35,36].

The clinical consequences of this gap are direct: patients who are discharged from inpatient rehabilitation before achieving functional independence—a group that constitutes a substantial proportion of stroke survivors—are precisely those who could benefit most from accessible, home-deployable mHealth support, and precisely those for whom such tools are currently least available. The underrepresentation of early rehabilitation in the mHealth literature likely reflects practical rather than conceptual barriers. Patients in the early subacute phase frequently present with concurrent motor, cognitive, and attentional impairments that complicate technology engagement, and safety requirements for unsupervised home-based exercise in this population are more demanding than in the chronic phase [37]. These barriers are real but not insurmountable: Burgos et al. [15] successfully enrolled participants at six to eight weeks post-stroke by incorporating caregiver co-training and remote safety monitoring into the intervention architecture. Future mHealth development for early rehabilitation should adopt analogous co-design principles from the outset.

The present review builds upon and diverges from a growing body of domain-specific reviews in meaningful ways. Thompson et al. [9] conducted a scoping review of mHealth interventions for post-stroke self-management support and identified inconsistent theoretical grounding across the literature; however, their scope was restricted to post-stroke self-management and RCT-evaluated interventions, excluding the acute diagnostic and workflow coordination tools that constitute six of the seventeen studies included here. Bonura et al. [10] provided an updated narrative review of smartphone applications in stroke management with a primary focus on acute-phase applications, while Kheirollahzadeh et al. [12] examined digital health technologies for at-home stroke recovery, restricting their scope to the post-discharge home recovery context. Yu et al. [13] examined implementation determinants of mHealth interventions for stroke recurrence prevention, offering a valuable framework for understanding low-resource barriers but addressing only secondary prevention rather than the full continuum.

In contrast to these domain-specific analyses, the present review adopts the stroke care continuum as its primary organizational framework, enabling a direct, phase-by-phase comparison of mHealth research activity across the full spectrum of stroke care. This integrative perspective reveals structural features that are invisible when any single phase is analyzed in isolation: specifically, the silo effect described above, the systematic underrepresentation of the early rehabilitation phase relative to both its clinical priority and the volume of research activity in adjacent phases, and the phase-constrained alignment of technology modalities. The continuum lane diagram (Figure 2) and evidence map (Figure 3) generated in this review provide complementary visual indices of this structure—the former exposing the temporal gaps across the care trajectory, the latter revealing the functional clustering of interventions within a narrow set of domain–technology combinations.

The present review also differs from prior work in its eligibility criteria. By requiring that the mHealth platform directly perform or deliver diagnostic, therapeutic, or rehabilitative functions—rather than serve solely as a communication or support tool—we applied a more stringent definition than several antecedent reviews. This decision improves construct validity by ensuring that included studies evaluate platforms with direct clinical functionality, but may have contributed to the low representation of secondary prevention, where many mHealth tools function primarily as medication reminder or behavioral coaching platforms.

The evidence map (Figure 3) reveals a strong and consistent alignment between technology modality and clinical domain that reflects functional necessity. In the acute diagnosis domain, all six included studies employed smartphone-based platforms, reflecting the unique combination of portability, wireless connectivity, and high-resolution imaging capability that smartphones offer in time-pressured clinical settings. This uniformity has a practical implication for clinical implementation: stroke systems of care contemplating mHealth integration in the acute phase can draw on a relatively homogeneous evidence base with consistent technology requirements, facilitating standardization of protocols and clinical training.

The rehabilitation domain exhibits considerably greater technology heterogeneity, with smartphones (*n* = 7), wearable devices (*n* = 1), and mixed-modality systems (*n* = 2) all represented across the ten included rehabilitation studies. This diversity reflects the multidimensional nature of post-stroke rehabilitation outcomes—motor performance, gait, balance, speech fluency, and cognitive function each impose different technological requirements—and the varied ecological settings in which rehabilitation is delivered (clinic, home, community). Mixed-modality systems, combining smartphone-delivered therapeutic content with wearable-enabled motion monitoring, were employed by Burgos et al. [15] and Pimentel et al. [29] and represent the most technically sophisticated paradigm in the reviewed literature. This trajectory toward sensor-enriched rehabilitation platforms enables both active therapeutic engagement and passive performance monitoring, without requiring patients to manage separate devices.

The secondary prevention domain presented the most striking disproportion: only one study—Ifejika et al. [20], a pilot RCT of a weight management smartphone application in obese minority stroke survivors—addressed secondary prevention, despite stroke recurrence rates of approximately 10% within the first year after index stroke [38,39]. The paucity of mHealth research in secondary prevention within this review partly reflects our stringent eligibility criteria, which excluded platforms functioning primarily as educational or reminder tools. Nevertheless, within the scope of functionally active mHealth interventions included in this review, the finding underscores an important research priority: the development and evaluation of mHealth platforms that directly support behavioral risk factor modification, physiological monitoring, and medication adherence in stroke survivors, using designs that advance beyond pilot feasibility toward adequately powered effectiveness trials.

The methodological profile of the included studies raises important questions about the readiness of the field for clinical implementation. Ten of the seventeen studies were interventional—five RCTs and five non-randomized studies—while seven were observational, comprising four cohort studies and three diagnostic accuracy studies. However, the median sample size across all seventeen studies was only 37 participants. Thirteen studies enrolled fewer than 50 participants, and four enrolled fewer than 15, a scale appropriate for proof-of-concept evaluation but insufficient for evidence synthesis or clinical guideline development. Statistical power is rarely explicitly reported in the included literature, and the small sample sizes make it likely that most published findings are insufficiently powered to detect clinically meaningful effect sizes, particularly for stroke rehabilitation outcomes where between-group differences are typically modest.

An encouraging temporal pattern is the acceleration in publication activity over the review period. Six studies were published in 2020, representing the baseline of the evidence base, but seven of the 17 studies (41%) were published in 2024 or 2025, suggesting rapidly growing research momentum. Several of the most recent studies demonstrate more rigorous design features: Pimentel et al. [29] employed a non-randomized controlled design with a validated wearable feedback system; Ruiz Ares et al. [30] evaluated a digital speech rehabilitation tool with standardized outcome measurement in an acute-to-subacute stroke population; and Kim et al. [21] conducted a fully virtual RCT for post-stroke aphasia rehabilitation with pre-specified primary outcomes. These design advances provide early evidence that the field is maturing toward clinical translation. The central challenge for the next generation of mHealth stroke research will be to sustain this methodological progression, moving from small-scale exploratory studies toward adequately powered confirmatory trials with standardized outcome measures and clearly articulated implementation pathways.

For clinicians working within stroke care systems, the findings of this review suggest several practical considerations. In the acute diagnostic setting, smartphone-based platforms for imaging interpretation and stroke team coordination represent the most mature mHealth application in stroke care. The diagnostic accuracy data from Komatsu et al. [22], Maxin et al. [26], and Maxin et al. [28] collectively indicate that smartphone-assisted imaging review achieves near-equivalent performance to conventional workstation-based assessment, with clinically acceptable interpretation times, supporting deployment in hub-and-spoke telemedicine models serving geographically dispersed populations.

For rehabilitation specialists, the evidence supports smartphone-based platforms as the most versatile and accessible technology for chronic-phase motor rehabilitation, given their ubiquity, low cost, and ease of integration into home programs. Mixed-modality systems incorporating motion-sensing wearables offer additional monitoring capability for balance and gait training, as demonstrated by Burgos et al. [15] and Pimentel et al. [29], though their implementation complexity and cost requirements are higher. The choice of technology platform should be guided by the specific therapeutic target and the patient’s cognitive and motor capacity for device operation, rather than by technology availability alone.

Most urgently, stroke care teams should recognize the current absence of evidence-based mHealth tools for the early rehabilitation phase as a clinical gap requiring proactive attention. In the absence of validated solutions for this transitional period, clinicians should consider adapting elements of existing chronic-phase platforms for early subacute use while actively advocating for and participating in clinical research targeting this underserved window. At the system level, healthcare administrators should note the geographic concentration of mHealth stroke research in high-income countries, with eight of seventeen studies (47%) conducted in the United States. The potential of mHealth to extend stroke care access in low- and middle-income settings—where stroke burden is disproportionately high and specialist rehabilitation resources are scarce—remains largely unexplored in the current evidence base. Investment in context-adapted mHealth development and evaluation in these settings represents both a scientific priority and an equity consideration [40,41].

## 5. Limitations

Several limitations should be considered when interpreting these findings. First, the search strategy did not include application-specific terms such as “mobile application” or “app-based,” which may have led to the under-identification of relevant studies, particularly consumer-facing interventions in rehabilitation and secondary prevention where app-based solutions are widely used but not always labeled as mHealth in the indexed literature. This limitation is especially likely to have affected the secondary prevention domain and should be addressed in future reviews through more granular search string construction. Second, no formal protocol was registered prior to initiation, although PRISMA-ScR guidelines were followed throughout; this is acknowledged as a methodological limitation. Third, our eligibility criteria required that the mHealth platform directly perform or deliver diagnostic, therapeutic, or rehabilitative functions, excluding platforms functioning primarily as communication, education, or monitoring tools. While this improves construct validity, it introduces a selection effect that may have further reduced the secondary prevention evidence base. Fourth, mobile stroke units and mobile CT-based prehospital strategies represent important diagnostic innovations, but were outside the scope of this review because they are infrastructure-based service models rather than application-, wearable-, or tablet-based mHealth interventions. Fifth, consistent with scoping review methodology, no formal quality appraisal was conducted; findings therefore reflect the distribution of research activity rather than the strength of the evidence. Fifth, the restriction to English-language publications may have introduced language bias, potentially underrepresenting mHealth research from non-English-speaking settings, particularly East and South Asia and Sub-Saharan Africa, where stroke burden is growing rapidly. Finally, studies published after the March 2025 search date were not captured; given the acceleration in publication activity observed over the review period, the current evidence base may underestimate the scope of ongoing research, particularly in areas such as AI-enabled diagnostic support and wearable-integrated rehabilitation platforms.

## 6. Conclusions

This scoping review mapped mHealth interventions across the stroke care continuum and identified a field that is growing in volume but remains structurally fragmented, phase-specific, and concentrated within a narrow set of clinical domains. Of the 17 included studies, 10 addressed rehabilitation—predominantly motor rehabilitation in the chronic phase—and six addressed acute diagnosis, while only one addressed secondary prevention. No study spanned more than a single phase of the stroke care continuum. An important gap is the near-absence of mHealth research in the early rehabilitation phase, the period of maximum neuroplasticity, despite its recognized importance for long-term functional recovery. Three priorities for future research emerge from these findings. First, mHealth intervention development should extend beyond the current phase-specific paradigm toward tools designed around the full patient trajectory, with particular urgency in the early rehabilitation phase and across the acute-to-community transition period. Second, research should expand beyond motor rehabilitation and acute diagnosis to include cognitive, linguistic, behavioral, and secondary prevention domains, which collectively account for a substantial burden of post-stroke disability and recurrence risk but remain underrepresented in the current evidence base. Third, the field must advance from small-scale feasibility studies—the majority of which enrolled fewer than 50 participants—toward adequately powered, rigorously designed trials that can generate evidence sufficient for clinical guideline development and implementation.

Achieving these goals will require coordinated investment in mHealth research across the full stroke care continuum, with particular attention to the transitional phases and underserved populations in low- and middle-income settings where the potential impact of mobile health technology is greatest. The continuum framework and evidence map presented in this review provide a structural foundation for identifying where this investment is most needed and for planning the next generation of integrated mHealth solutions for stroke care.

## Figures and Tables

**Table 1:** Studies of mobile health interventions for stroke care.

Author (Year)	Country	Stroke Stage	Stroke Type	Care Domain	Study Design	Sample Size
Burgos et al. (2020) [15]	Chile	Subacute	Both included	Rehabilitation	Interventional (RCT)	10
Chen et al. (2024) [16]	USA	Chronic	Not specified	Rehabilitation	Interventional (non-randomized)	10
Cordella et al. (2022) [17]	USA	Mixed	Not specified	Rehabilitation	Observational (cohort)	2249
Grau-Pellicer et al. (2020) [18]	Spain	Chronic	Both included	Rehabilitation	Interventional (RCT)	41
Hutchinson et al. (2020) [19]	USA	Chronic	Not specified	Rehabilitation	Interventional (non-randomized)	11
Ifejika et al. (2020) [20]	USA	Subacute	Both included	Secondary prevention	Interventional (RCT)	36
Kim et al. (2025) [21]	Canada	Chronic	Not specified	Rehabilitation	Interventional (RCT)	37
Komatsu et al. (2021) [22]	Japan	Acute	Ischemic only	Acute Diagnosis	Diagnostic accuracy	108
Langan et al. (2020) [23]	USA	Chronic	Not specified	Rehabilitation	Interventional (non-randomized)	16
Mansour et al. (2021) [24]	Egypt	Acute	Ischemic only	Acute Diagnosis	Observational (cohort)	360
Martins et al. (2020) [25]	Brazil	Acute	Ischemic only	Acute Diagnosis	Observational (cohort)	442
Maxin et al. (2024) [26]	USA	Acute	Hemorrhagic only	Acute Diagnosis	Diagnostic accuracy	49
Maxin et al. (2025) [27]	USA	Acute	Ischemic only	Acute Diagnosis	Observational (cohort)	22
Maxin et al. (2025) [28]	USA	Acute	Both included	Acute Diagnosis	Diagnostic accuracy	33
Pimentel et al. (2025) [29]	Brazil	Chronic	Both included	Rehabilitation	Interventional (non-randomized)	40
Ruiz Ares et al. (2025) [30]	Spain	Acute	Both included	Rehabilitation	Interventional (non-randomized)	40
Toh et al. (2025) [31]	China	Chronic	Not specified	Rehabilitation	Interventional (RCT)	12

Abbreviation: RCT, randomized controlled trial.

**Table 2:** Mobile health technologies used across stroke care domains.

Care Domain (*n* ^a^)	Smartphone	Wearable	Tablet	Mixed
Acute diagnosis (6)	●●●●●●			
Rehabilitation (10)	●●●●●●	●	●	●●
Secondary prevention (1)	●			

^a^ Numbers in parentheses indicate the number of included studies. Filled circles represent individual studies within each technology category.

## Data Availability

No new data were created or analyzed in this study. Data sharing is not applicable to this article.

## Supplementary Materials

The following supporting information can be downloaded at: https://www.mdpi.com/article/10.3390/jcm15114121/s1, Table S1. PRISMA-ScR-Checklist.

## Abbreviations

The following abbreviations are used in this manuscript:

DOI	digital object identifier
mHealth	mobile health
RCT	randomized controlled trial
PRISMA-ScR	preferred reporting items for systematic reviews and meta-analyses extension for scoping reviews
